# Evaluation of Antioxidant and Anti-Inflammatory Activities, and Metabolite Profiling of Selected Medicinal Plants of Nepal

**DOI:** 10.1155/2023/6641018

**Published:** 2023-11-03

**Authors:** Amit Kumar Shrivastava, Muskan Keshari, Manisha Neupane, Sheshbhan Chaudhary, Purna Kala Dhakal, Laxmi Shrestha, Anjan Palikhey, Chandrajeet Kumar Yadav, Gopal Lamichhane, Mohammad Ujair Shekh, Rakesh Kumar Yadav

**Affiliations:** ^1^Department of Pharmacology, Universal College of Medical Sciences, Bhairahawa, Rupandehi 32900, Nepal; ^2^Department of Pharmacy, Universal College of Medical Sciences, Bhairahawa, Rupandehi 32900, Nepal; ^3^Department of Nutritional Sciences, Oklahoma State University, Stillwater, OK 74078, USA; ^4^School of Health and Allied Sciences, Pokhara University, Pokhara-30, Kaski, Nepal; ^5^Institute of Agriculture and Animal Science, Tribhuvan University, Paklihawa, Rupandehi, Nepal

## Abstract

This study aimed to evaluate the antioxidant, antiarthritic, and anti-inflammatory properties of extracts from the leaves of twelve different medicinal plants in Nepal. We then evaluated the total phenolic, flavonoid, and tannin contents of the extract using in-vitro assays and characterized it using GC-MS analysis. Results revealed that most of the leaf extracts contained phenolic compounds, flavonoids, tannins, alkaloids, and saponins. Few plants also showed the presence of glycosides, phytate, and vitamin C. Among the studied plants, *Neolamarckia cadamba* exhibited the highest total phenolic and tannin contents, as 241.53 ± 0.20 *µ*g of gallic acid equivalent/mg and 74.48 ± 1.081 *µ*g of tannic acid equivalent/mg, respectively. *Ipomoea batatas* exhibited the highest total flavonoid content, as 53.051 ± 1.11 *µ*g of quercetin equivalent/mg. Moreover, *Raphanus sativus* demonstrated significant ferrous ion chelating, 2,2-diphenyl-1-picrylhydrazyl, hydrogen peroxide scavenging, and total antioxidant activities with IC_50_ value of 4.76 ± 0.68 *µ*g/mL, 5.84 ± 0.14 *µ*g/mL, 6.89 ± 0.16 *µ*g/mL, and 8.99 ± 0.20 *µ*g/mL, respectively. Similarly, *Colocasia esculenta* and *Cicer arietinum* exhibited the highest hydroxyl radical and nitric oxide scavenging activities, measuring IC_50_ value of 7.22 ± 0.56 *µ*g/mL and 9.06 ± 0.10 *µ*g/mL, respectively. Among all the extracts, *Amorphophallus paeoniifolius* displayed significant human red blood cell (HRBC) membrane stabilization activity (IC_50_ = 6.22 ± 0.78 *µ*g/mL). Furthermore, *Raphanus sativus*, *Chenopodium album, Cicer arietinum*, and *Murraya koenigii* exhibited the highest inhibitory activities against protein denaturation with bovine serum albumin, antiarthritic, lipoxygenase inhibitory, and proteinase inhibitory, measuring IC_50_ of 7.48 ± 0.48 *µ*g/mL, 9.44 ± 1.62 *µ*g/mL, 14.67 ± 1.94 *µ*g/mL, and 28.57 ± 2.39 *µ*g/mL, respectively. In conclusion, this study demonstrated the twelve leaf extracts' significant antioxidant, antiarthritic, and anti-inflammatory activities.

## 1. Introduction

Natural products have been utilized as herbal/natural medicine from ancient times and are a major option for healing for many of the population until today [1]. The knowledge that humans gathered from using herbal medicine from ancient times can be a good resource that increases the likelihood of discovering bioactive compounds and formulations [1]. This reason has made phytochemical screening from several natural products with an ethnomedicinal value to increase rapidly in these days [2]. Also, the relatively lower adverse effects and toxicity of herbal medicines compared to chemically synthesized drugs are the reason for the inclination towards herbal research [3]. Various active phytoconstituents were extracted from herbs and plants that prevent and cure chronic illnesses caused by oxidative stress. Many phytoconstituents were already reported to have antiarthritic, anti-inflammatory, and antioxidant activities [4].

As such, andrographolide is the biologically active diterpenoid lactone extracted from *Andrographis paniculata* (an ancient herb found in Asian countries) used to treat flu, upper respiratory tract, and sore throat [5]. They are multitarget medications with anti-inflammatory and antioxidant in various cell types [6]. Using multiple doses of 25, 50, and 100 mg/kg in rats, they found andrographolide to decrease the level of articular elastase, TNF-*α*, IL-6, CXC chemokine ligand 2, and myeloperoxidase and increase the concentration of catalase, superoxide dismutase, and glutathione (antioxidant enzymes) [7].

Inflammation is a defensive mechanism triggered by harmful foreign stimuli, such as pathogens, viruses, dust particles, irritants, and damaged cells, to initiate healing [8, 9]. It comprises various steps, starting with an induction phase, continuing with a peak of inflammation, and ending with the resolution phase [10]. Induction phase is needed for effective host defense. It is caused due to external and endogenous noxious stimuli resulting from mechanical, chemical, or biological cell destruction [11]. The resolution phase is necessary for reducing inflammation and restoring cell homeostasis after removing the noxious stimuli. Exaggerated immune responses such as human leukocyte antigen-B27 in arthritis, nucleotide-binding oligomerization domain-2, and interleukin-10-R mutations are hereditary factors that can override suitable resolution mechanisms [12].

The coordinated activity of mediators and effector cells causes inflammation. Cytokines are responsible for inflammation [13]. They are characterized as proinflammatory cytokines, namely, interleukin- (IL-) 1*β*, IL-6, and tumor necrosis factor- (TNF-) *α*, and the anti-inflammatory cytokines, namely, IL-1R*α*, IL-4, IL-10, and transforming growth factor-*β*1 [14]. Proinflammatory cytokines enhance systemic infection and initiate an immune response to disease, whereas anti-inflammatory cytokines counteract these effects to reduce inflammation and promote healing [15]. Also, inflammation promotes and regulates many biological responses such as apoptosis, necrosis, and autophagy caused due to oxidative stress [16]. Oxidative stress and inflammation are linked pathophysiological events; mechanisms are evident in various diseases, including irritable bowel syndrome, inflammatory bowel disease, and ulcerative colitis [17]. The presence of inflammatory cells leads to the nitration and oxidation of large molecules such as proteins, lipids, DNA, and RNA. This process generates numerous free radicals, including reactive oxygen species (ROS) [18]. The proinflammatory cytokines, such as IL-1, IL-18, TNF-*α*, and p38 mitogen-activated protein kinases (MAPK), are primarily involved in creating ROS, which leads to oxidative stress [19].

Increased burden of diseases and success story of identification of antioxidant and anti-inflammatory compounds in traditional herbs motivated us to evaluate antiarthritic, anti-inflammatory, and antioxidant activities on leaves of twelve different herbs, namely, *Amorphophallus paeoniifolius*, *Ipomoea batatas*, *Amaranthus viridis, Cicer arietinum, Murraya koenigii, Colocasia esculenta, Raphanus sativus, Neolamarckia cadamba, Dioscorea bulbifera, Chenopodium album, Cinnamomum tamala,* and *Brassica nigra,*.

This study aims to evaluate antioxidant, anti-inflammatory, and antiarthritic activities of twelve native medicinal plants found in Nepal and characterize their extract using the LC-MS technique. This study helps to understand the biological activities of these plants and identify bioactive compounds responsible for those activities.

## 2. Materials and Methods

The experimental study was conducted in the Department of Pharmacology, Universal College of Medical Science (UCMS), Bhairahawa, Rupandehi, from December 2021 to May 2022. Before starting this study, the project registration was completed by the Institutional Review Committee of Universal College of Medical Sciences, Bhairahawa, Rupandehi, Nepal Registration Number: UCMS/IRC/032/22.

### 2.1. Plant Material Collection and Verification

In the present study, most of medicinal plants used were seasonal; therefore, the fresh leaves of all those twelve medicinal plants, namely, *Cicer arietinum, Murraya koenigii, Colocasia esculenta, Amorphophallus paeoniifolius, Ipomoea batatas, Amaranthus viridis, Raphanus sativus, Neolamarckia cadamba, Dioscorea bulbifera, Chenopodium album, Cinnamomum tamala,* and *Brassica nigra,* were collected from Rupandehi district in December 2021. The plants were verified and certified by Mr. Subodh Khanal, Assistant Professor and Course In-charge of Medicinal and Aromatic Plants, Department of Soil and Environmental Science, Institute of Agriculture and Animal Science (IAAS), Paklihawa, Rupandehi, Nepal. The specimen number of individual plants is listed in Table 1.

### 2.2. Plant Extraction Process

First, the fresh leaves of all twelve plants were washed with fresh water to remove the dust; after that, the fresh leaves of individual plants were introduced with liquid nitrogen separately to perform the freeze-drying process. 5 mL of ethanol (70% v/v) was mixed with one gram of freeze-dried sample for about five minutes in a magnetic stirrer at high speed, followed by centrifugation at 4500 rpm for 10 minutes. After that, the supernatant was stored and filtered with the help of filter paper. The remaining residue was re-extracted following the same procedures, and the collected supernatants were combined. Then, the combined supernatant was dried in a rotatory evaporator at 40°C. The dried extract was accurately weighed and stored at −20°C in an airtight screw-capped glass bottle and was used for assay by dissolving the extract in ethanol in a required concentration [20].

(1) $$\begin{array}{c} \%\text{ yield}=\frac{\text{Weight of extract obtained }\left(g\right)}{\text{Weight of plant powder extracted }\left(g\right)}\times 100. \end{array}$$

### 2.3. Determination of Phytochemical Constituents

#### 2.3.1. Determination of Moisture Content

The sample was exactly weighed before and after oven drying to measure the loss on drying. Moisture content (MC) was calculated as follows [21]:

(2) $$\begin{array}{c} \text{Moisture content on }a\text{ wet basis }\left(\%\text{ moisture}\right)=\frac{\text{wt of wet sample }\left(g\right)\;–\text{ wt of dry sample }\left(g\right)}{\text{wt of wet sample }\left(g\right)}\times 100\%. \end{array}$$

#### 2.3.2. Estimation of Chlorophyll Content

Some fresh leaves of selected plants were taken and cut individually. The chopped leaves of those plants were accurately weighed (1 g) and grounded using a mortar pestle separately. Then, 20 mL of 80 percent acetone and 0.5 g of magnesium carbonate powder were added. The leaves were again appropriately ground for 4 minutes at 40°C, and the samples were put into the refrigerator for the next 24 hours. After 24 hours, the samples were centrifuged at 500 rpm for 5 minutes. The supernatants were transferred to 100 mL of volumetric flask, and 80% acetone was added to the prepared solution to maintain the final volume. The absorbance was measured using a spectrophotometer at 645 and 663 nm [20].

#### 2.3.3. Preliminary Phytochemical Screening of Different Plant Extracts

The preliminary phytochemical screening of various plant extracts was carried out using the standard procedure mentioned in previous studies. Test for alkaloids are as follows:: Test with Dandruff reagent [22], test with Mayer's reagent, test with Wagner's reagent, and test with Hager's reagent [23]. Estimation of saponins are as follows: Foam test [24] and determination of phytate content [25].

#### 2.3.4. Determination of Total Phenolic Content (TPC)

The method explained by Hazra et al. was used for the determination of total phenolic content by using the Folin–Ciocalteu phenol reagent [24]. A total of 2 mL of plant extract (100–2000 *μ*g/mL) and 2 mL of Folin–Ciocalteu phenol reagent were mixed, and the solution was made 9 mL each with distilled water. The solution of sodium carbonate was added [1 mL of 10% (w/v)] after 5 minutes and mixed thoroughly. The total 10 mL of the reaction mixtures were kept for 1 hour at room temperature, and the absorbance was measured using a UV spectrophotometer at 765 nm. Using an equation derived from the gallic acid standard calibration curve, the total phenolic content for each extract was expressed in milligrams of gallic acid equivalent per milligram of extract (a µg of GAE/mg of extract). The entire experiment was carried out in triplicate [26].

#### 2.3.5. Determination of Total Flavonoid Content

The total flavonoid content for all the twelve plant extract samples was determined using the aluminum chloride colorimetric method, previously explained by Chang et al. 2002. The solution was separely prepared containing 1000 µL quercetin and plant extracts at different concentrations (100-2000 µg/mL), 200 *µ*L potassium acetate (1 M, merk), 3000 *µ*L ethanol (95 percent), 200 µL of 10% aluminium chloride and 5600 *µ*L distilled water, the reaction mixture was kept at room temperature for 30 minutes. After that absorbance was measured at 412 nm using a spectrophotometer. Flavonoid content in each sample was evaluated using a standard calibration curve of quercetin and expressed in terms of the µg quercetin equivalent per milligram (µgQAE/mg) of extract. The experiments were all carried out in triplicate [27].

#### 2.3.6. Determination of Tannin Content

It was based on the Haq et al. method. Tannic acid was taken as the standard to detect the tannin content. Different concentrations (100-2000 µg/mL) of plant extracts (500 *µ*L) were taken in a test tube with distilled water (500 *µ*L) and 100 mg polyvinylpolypyrrolidone (PVP) and incubated at 4°C for 4 hours. Then, it was centrifuged at 5000 rpm for 5 minutes, and 20 *µ*L supernatant phenolic content was taken (free from tannins due to precipitation formation with PVP). Absorbance was determined at 725 nm and expressed in terms of *µ*g tannic acid equivalent per milligram (µg TAE/mg extract) using a standard curve [28].

### 2.4. Gas Chromatography and Mass Spectroscopy (GC-MS)

In this study, we performed GC-MS spectroscopic analysis of ethanolic leaf extracts to determine their phytoconstituents. GC-MS extract analysis was conducted at the Nepal Academy of Science and Technology (NAST), Lalitpur, Kathmandu, Nepal. The analysis was carried out by using Agilent 7890A spectrometer system with fused silica column Agilent 19091s-433 (5% phenyl methyl siloxane 30 m × 250 *µ*m, film thickness × 0.25 *µ*m), interfaced with GC-Agilent 5975C inert MSD with triple axis detector. In this spectroscopy, helium gas was used as carrier gas with a flow speed of 1 mL/minute. The spectrophotometer ion source temperature was 230°C, with an interface temperature was 320°C, pressure 6.6018 psi, and the injector volume was 2 *µ*L in split mode with a ratio of 75 : 1 (75 mL/minutes) at a temperature of 320°C. Initially, the spectrophotometer column temperature was started at 32°C for 5 minutes. After that, it was changed to 70 eV and ion source temperature 280°C in the scan range of 50-100 amu for 5 minutes. The total run time of individual samples was 59.6 minutes. After the analysis, the relative percent amount of each component was calculated by comparing the average peak % area [29].

### 2.5. *In-Vitro* Antioxidant Activity

The antioxidant activity of twelve plant extracts was estimated by using various methods, including (2, 2-diphenyl-1-picrylhidrazyl) DPPH free radical scavenging activity [30, 31], ferrous (Fe^2+^) ion chelating agent [32], ferric (Fe^3+^) ion reducing power [24], nitric oxide (NO^−^) scavenging activity [33], hydroxyl radical scavenging activity [34], hydrogen peroxide scavenging activity [35], and total antioxidant activity [30].

### 2.6. *In-Vitro*Anti-Inflammatory Activity and Antiarthritic Activity

#### 2.6.1. Protein Denaturation Assay

We used the method explained by Palit and his colleagues to determine the antiarthritic activity, with slight modification [36]. 0.10 mL of different plant leaf extract dissolved in DMSO was used separately at 100 − 2000 *μ*g/mL concentration. The solution mixture was again mixed with 2.40 mL of 3.5% bovine serum albumin (BSA) solution. The 1N of hydrochloric acid at pH 6.3 was prepared and added to the extract solution. After that, the solution was incubated at 37°C for 20 minutes. The reaction mixture was then heated at 71°C for 1 minute. After cooling solution, 2.5 mL of phosphate-buffered saline (PBS) with pH 6.3 was added to individual samples. The absorbance of the final reaction mixture was measured at 660 nm by using a UV spectrophotometer. Product control was prepared with BSA, and the percentage of protein denaturation inhibition will be estimated by using the following formula:

(3) $$\begin{array}{c} \%\text{ inhibition}=\left[1-\frac{\text{Abs of test}-\text{Abs of control}}{\text{Abs of control}}\right]\times 100, \end{array}$$

where test is mean absorbance of plant extract and reagents and control is mean absorbance of solvents and reagents without inhibitor.

#### 2.6.2. Membrane Stabilization

*(1) Erythrocyte Suspension*. With few modifications, the erythrocyte suspension was prepared using the technique outlined by Pieroni et al. [37]. Healthy human volunteers provided the blood sample, which was centrifuged for 10 minutes at 1500 rpm. The red blood cell (RBC) pallet was washed three times with PBS (pH 7.4) following centrifugation. The buffy coat and supernatant liquid were aspirated out following each wash. The RBCs were resuspended in PBS at a concentration of 20% (v/v).

*(2) Heat-Induced Hemolysis*. The method explained by Gunathilake et al. was used to determine the heat-induced hemolysis with some modifications. A 0.05 mL at varying concentrations of 100 − 2000 *μ*g/mL of hydroethanolic extract of different plant leaves was taken separately, and a 0.05 mL of blood erythrocyte suspension was mixed with 2.95 mL of PBS at pH value 7.4. Then, the mixture was incubated for 20 minutes at 54°C in the water bath with continuous shaking. The mixture was incubated and centrifuged at 2500 rpm for 3 minutes. By using a UV spectrophotometer, the absorbance of supernatant was measured at 540 nm. In the experiment, PBS will be used as blank [38]. The hemolysis level will be calculated using the following equation:

(4) $$\begin{array}{c} \%\text{ inhibition}=\left[1-\frac{\text{Abs of test}-\text{Abs of control}}{\text{Abs of control}}\right]\times 100. \end{array}$$

*(3) Proteinase Inhibitory Activity*. The method explained by Truong et al. was used to evaluate the proteinase inhibitory activity of plant extract with slight modification [38]. A 0.06 mg trypsin and 1 mL of 20 mM Tris-HCl buffer with pH 7.4 were mixed with 1 mL of individual plant extract at 100 − 2000 *μ*g/mL concentration. The mixture was incubated at 37°C for 5 minutes, and then, 1 mL of 0.7% (w/v) casein protein was added. Again, the reaction mixture was incubated for 20 minutes. Then, 1 mL of 70% perchloric acid was added to end the reaction. The reaction mixture was centrifuged at 3000 rpm at 4°C for 10 minutes. The absorbance of the supernatant will be measured at 210 nm against the buffer solution as blank by using a spectrophotometer.

(5) $$\begin{array}{c} \%\text{ inhibition}=\left[1-\frac{\text{Abs of test}-\text{Abs of control}}{\text{Abs of control}}\right]\times 100. \end{array}$$

#### 2.6.3. Lipoxygenase Inhibition Assay

The method explained by Wu et al. was used to evaluate the lipoxygenase inhibition activity of plant extract with some modifications [39, 40]. The linoleic acid was used as substrate, and the 5-lipoxidase enzyme was used. The solution mixture containing 1 mL of sodium borate buffer (0.1M) with pH 8.8 and 10 *µ*L lipoxygenase (final concentration 8000 U/mL) was incubated with 1 mL plant extract at a concentration of 100 − 2000 *μ*g/mL. The reaction mixture was incubated at room temperature at 30 ± 2°C for 5 minutes. The 10 *µ*L linoleic acid (10 mmol) was added and initiated the reaction. By using a UV spectrophotometer, the absorbance was calculated at 234 nm. The % inhibition of lipoxygenase will be calculated by using the following formula:

(6) $$\begin{array}{c} \%\text{ inhibition}=\left[1-\frac{\text{Abs of test}-\text{Abs of control}}{\text{Abs of control}}\right]\times 100. \end{array}$$

### 2.7. Statistical Analysis

The resultswere expressed as mean ± standard error mean (SEM). Data analysis was performed using GraphPad Prism Version 5 and 8.0. The mean was compared by one-way analysis of variance followed by Tukey's multiple comparison test. The values were statically significant at three levels, ^*∗∗∗*^*p* < 0.001, ^*∗∗*^*p* < 0.01, and ^*∗*^*p* < 0.05 but nonsignificant (NS) if *p* > 0.05.

## 3. Results

### 3.1. Percentage Yield

An extraction technique is considered ideal when it produces high yields of extracts while requiring little modification to the extract's functional qualities [37]. The difference in the efficacy of biological activities was observed in extracts generated using various extraction procedures [41]. As a result, it is important to choose the best extraction technique and solvent based on the sample matrix's characteristics, the analytes' chemical properties, the interaction of the matrix and the analytes, and the effectiveness and desired attributes [37]. Extraction yields were determined by evaporating the different ethanolic extracts obtained in the ratio 1 : 5 of extract and solvent at 37°C in an incubator to dryness and measuring the remaining solid residue. The extractive yield of each ethanolic leaf extract was determined and stated in Table 1. From the above table, *Neolamarckia cadamba* has the highest yield percentage, and *Colocasia esculenta* has the lowest yield percentage, which was found to be 17.62% and 7%, respectively.

### 3.2. Preliminary Phytochemical Screening

The preliminary phytochemical screening of twelve plant leaf extracts showed that *C. arietinum, M. koenigii, A. viridis, A. paeoniifolius,* and *N. cadamba* showed the presence of a high number of bioactive metabolites, and the remaining other plants leaves extract also showed good but not similar bioactive secondary metabolites profile. The absence and presence of different secondary metabolites were identified by performing different tests and reported the presence (+) and absence (−) in the form of symbols (Table 2).

### 3.3. Moisture and Chlorophyll Content

Table 1 shows the data of the combined chlorophyll and moisture content. All plant leaves were incubated at 37°C, and it was found that *Amaranthus viridis* exhibited the highest moisture content (2.82 ± 0.28 mg/mL), while *Chenopodium album* exhibited the lowest (0.24 ± 0.001 mg/mL). *Amorphophallus paeoniifolius* had the highest chlorophyll content, measuring 13.59 ± 0.003, while *Cicer arietinum* had the lowest, measuring 0.10 ± 0.035, respectively.

### 3.4. Total Phenolic Content (TPC)

The phenolics are the important phytoconstituents having redox properties responsible for scavenging free radicals [42]. In present studies, the total phenolic content of different ethanolic plant extracts weredetermined using the Folin–Ciocalteu assay by creating a standard calibration curve with gallic acid into the relationship between absorbance and concentration (*y* = 0.0098*x* + 0.0058 and *R*^2^ = 0.995). Using the equation derived from the calibration curve of gallic acid, the difference in total phenolic content was large among all extracts, ranging from 25.71 ± 0.49 to 241.53 ± 0.20 *µ*g GAE/mg of extract. The results showed that *N. cadamba* had the highest total phenolic content 233.19 ± 8.69 *µ*g GAE/mg, followed by *D. bulbifera* 201.22 ± 4.92, *C. arietinum* 157.17 ± 0.70, *C. tamala* 164.14 ± 3.83, A*. viridis* 162.61 ± 3.48, *B. nigra* 163.63 ± 1.07, *R. sativus* 151.9 ± 1.06, *A. paeoniifolius* 100.71 ± 1.35 *μ*g GAE/mg, respectively, and so on at 1000 *μ*g/mL concentration. It suggested that total phenolic content was superlatively extracted through ethanol; increasing the concentration of plant extract knowingly increased the total phenolic content that is expressed in supplementary Table 1.

### 3.5. Total Flavonoid Content

Flavonoids are the secondary metabolites of medicinal plants including flavones, flavanols, and condensed tannins. They have shown good antioxidant properties attributed mainly to the presence of free hydroxyl groups [42]. In the present study, the total flavonoid contents of different ethanolic extracts were determined using calibration curve of quercetin, comparing absorbance with that of crude extracts (*y* = 0.0472*x* + 0.0061 and *R*^2^ = 0.9984). Supplementary Table 2 represents the total flavonoid content of different plant extracts of concentration 100 − 2000 *μ*g/mL expressed as in quercetin *μ*g QAE/mg of dried extract weight. Among these mentioned crude plant extracts, *I. batatas* had the highest total flavonoid content 51.82 ± 1.47 *µ*g of QAE/mg of extract followed by *C. esculenta* 40.58 ± 5.40, *C. album* 44.61 ± 0.24, *A. paeoniifolius* 35.96 ± 0.16, *D. bulbifera* 34.55 ± 1.48, *N. Cadamba* 33.74 ± 0.12 *μ*g QAE/mg, respectively, and so on at 1000 *μ*g/mL concentration.

### 3.6. Total Tannin Content

In the present study, the total tannin contents of different ethanolic extracts were determined using a calibration curve oftannic acid and comparision of absorbance of crude extracts (*y* = 0.0154*x* + 0.0033 and *R*^2^ = 0.9968). Using the equation derived from the calibration curve of tannic acid, the total tannin contents range from 31.62 ± 1.117 to 74.48 ± 1.081 *µ*g of TAE/mg of extract. Among these mentioned crude extracts, *N. cadamba* contains the highest total tannin content, 72.64 ± 1.60 *µ*g TAE/mg, followed by *D. bulbifera* 71.88 ± 0.86, *B. nigra* 66.36±2.27, *R. sativus* 66.14 ± 2.16, *C. tamala* 61.16 ± 3.25, *C. albumμ*g TAE/mg, respectively, and so on at 1000 *μ*g/mL concentration. Whereas increasing the extract concentration, the total tannin content was also increased (supplementary Table 3).

### 3.7. GC-MS Analysis

The present study used GC-MS analysis to determine the phytoconstituents present in ethanolic extract of twelve medicinal plants. In the GC-MS analysis, the compounds were predicted by calculating the spectrum's average peak percentage area and retention time. All the phytoconstituents present in several plant leaf extracts are listed in Table 3. Also, the mass spectrophotometer determines the compounds eluted at different time frames and helps to identify the compounds' nature and structure (supplementary Figure 1). The GC-MS analysis helps predict the phytoconstituent present in different leaves plant extract. It may lead to the isolation of those phytoconstituents present in high concentration into the specific plant extract. Similarly, determining their pharmacological activity will be helpful for the discovery of new molecules.

### 3.8. *In-Vitro* Antioxidant Activity

The antioxidant activity of ethanolic extract (100 − 2000 *μ*g/mL) from twelve different plants was carried out by taking ascorbic acid, BHT, and BHA, as standard compounds. The plant extracts were tested against the oxidant compound for showing significant antioxidant properties. The plant extracts were tested by taking different concentrations and found that the activity was concentration-dependent, i.e., increment in the concentration of plant showed better antioxidant activity and vice versa. Almost all plant extracts showed significant antioxidant activity.  Table 4 indicates that lower IC_50_ values have more inhibitory activity. The standard compound ascorbic acid has shown more antioxidant activity than the other standard compounds, including BHA in nitric oxide scavenging activity and BHT in H_2_O_2_, DPPH, and hydroxyl scavenging activity and ferrous ion chelating activity. Among all the plant extracts, *R. sativus*, *C. esculenta*, *C. arietinum*, and *I. batatas* showed significantly highest antioxidant activity. The plant extract of *R. sativus* is most active for showing ferrous ion chelating activity, DPPH and H_2_O_2_ scavenging activity, and total antioxidant activity (IC_50_ 4.76 ± 0.68 *µ*g/mL, 5.84 ± 0.14 *µ*g/mL, 6.89 ± 0.16 *µ*g/mL, and 8.99 ± 0.20 *µ*g/mL), respectively. Also, the extract of *C. esculenta* shows better hydroxyl radical, H_2_O_2_, and DPPH scavenging activity, with IC_50_ 7.22 ± 0.56 *µ*g/mL, 7.56 ± 0.52 *µ*g/mL, and 8.91 ± 0.12 *µ*g/mL, respectively. Similarly, *C. arietinum* showed highest total antioxidant activity, nitric oxide, and DPPH scavenging activity (IC_50_ 8.38 ± 0.09 *µ*g/mL, 9.06 ± 0.10 *µ*g/mL, and 9.41 ± 0.07 *µ*g/mL, respectively), and the plant extract *C. album* and *I. batatas* show the highest DPPH (6.13 ± 1.23 *µ*g/mL) and H_2_O_2_ (6.63 ± 0.46 *µ*g/mL) scavenging activity, respectively. The least ferrous ion chelating activity, ferric ion reducing activity, and hydroxyl radical scavenging activity were demonstrated by *A. paeoniifolius* (IC_50_ 54.44 ± 3.68 *µ*g/mL), *M. koenigii* (IC_50_ 45.41 ± 1.65 *µ*g/mL), and *C. album* (IC_50_ 43.54 ± 0.19 *µ*g/mL), respectively. According to the table below, a similar type of H_2_O_2_ scavenging activity has been shown by plant extract of *N. cadamba* (IC_50_ 7.01 ± 0.14 *µ*g/mL) and *C. tamala* (IC_50_ 7.81 ± 1.8 *µ*g/mL). Also, the plant extracts of *B. nigra* and *A. viridis* (IC_50_ 8.44 ± 0.97 *µ*g/mL and 8.70 ± 0.39 *µ*g/mL) have similar DPPH scavenging activity.

The ferric ion-reducing antioxidant power assay of twelve plant extracts was carried out. The activity was confirmed when the color of the solution was changed from yellow to green and Prussian blue based on the reduction property of extracts and standard, which was measured at 700 nm wavelength using a UV spectrophotometer. The absorbance value was increased with the increasing concentration of plant extract (Table 4). So, the activity was found to be concentration-dependent.

### 3.9. *In-Vitro* Anti-arthritic and Anti-Inflammatory Activities

The antiarthritic and anti-inflammatory properties of ethanolic extract (100 − 2000 *μ*g/mL) of the plants *A. viridis, I. batatas*, *A. paeoniifolius*, *N. cadamba*, *R. sativus, C. arietinum*, *C. esculenta*, *M. koenigii*, *B. nigra*, *C. tamala,* and *C. album* were compared with the standard compound indomethacin, aceclofenac, etoricoxib, and aspirin. The values in the table are the IC_50_ values, indicating that the compound with less IC_50_ results in good inhibitory activity. Among the standard compounds, indomethacin responds better to all the protein denaturation activity with BSA, human red blood cells (HRBC) membrane stabilization, lipoxygenase (LOX), proteinase inhibitory and antiarthritic activity, and etoricoxib for protein denaturation with BSA and human red blood cells (HRBC) membrane stabilization, and aceclofenac for protein denaturation with BSA and lipoxygenase (LOX) inhibitory activity and aspirin for protein denaturation with BSA.

Among the plant extract, the best response of human red blood cells (HRBC) membrane stabilization was confirmed by *A. paeoniifolius* (IC_50_ 6.22 ± 0.78 *µ*g/mL), *I. batatas* (IC_50_ 10.26 ± 0.65 *µ*g/mL), and *N. cadamba* (IC_50_ 16.05 ± 2.67 *µ*g/mL), protein denaturation activity with BSA by the plant extract of *R. sativus* (IC_50_ 7.48 ± 0.48 *µ*g/mL) and *A. viridis* (IC_50_ 15.09 ± 1.94 *µ*g/mL), antiarthritic activity by the plant extract of *C. album* (IC_50_ 9.44 ± 1.62 *µ*g/mL) and *C. tamala* (IC_50_ 11.81 ± 2.51 *µ*g/mL), lipoxygenase (LOX) inhibitory activity by the plant extract of *C. arietinum* (IC_50_ 14.67 ± 1.94 *µ*g/mL) and *D. bulbifera* (IC_50_ 15.01 ± 0.23 *µ*g/mL), and proteinase inhibitory by plant extract of *M. koenigii* (IC_50_ 28.57 ± 2.39 *µ*g/mL), respectively*. C. album* (IC_50_ 70.72 ± 4.20 *µ*g/mL) and *M. koenigii* (IC_50_ 59.14 ± 2.85 *µ*g/mL) showed comparatively lower activity than other plant extracts for human red blood cells (HRBC) membrane stabilization activity (IC_50_ 70.72 ± 4.20 *µ*g/mL), LOX inhibition activity by the plant extract *C. tamala* (IC_50_ 68.16 ± 4.34 *µ*g/mL), *A. paeoniifolius* (IC_50_ 63.96 ± 3.99 *µ*g/mL)*, I. batatas* (IC_50_ 62.94 ± 3.53 *µ*g/mL) and *A. viridis* (IC_50_ 58.99 ± 3.84 *µ*g/mL), antiarthritic activity by *D. bulbifera* (IC_50_ 66.90 ± 4.03 *µ*g/mL) and *C. esculenta* (IC_50_ 56.2 ± 3.74 *µ*g/mL), proteinase inhibitory by plant extract of *R. sativus* (IC_50_ 65.52 ± 2.53 *µ*g/mL), protein denaturation with BSA by plant extract *N. cadamba* (IC_50_ 62.11 ± 2.50 *µ*g/mL), *C. arietinum* (IC_50_ 61.65 ± 2.67 *µ*g/mL) and *B. nigra* (IC_50_ 40.79 ± 3.19 *µ*g/mL), respectively. The plants extract of *B. nigra* (IC_50_ 13.1 ± 1.81 *µ*g/mL) and *C. esculenta* (IC_50_ 13.50 ± 2.94 *µ*g/mL) reported a similar type of proteinase inhibitory activity. Also, the plant extract of *N. cadamba* (IC_50_ 35.98 ± 2.99 *µ*g/mL) and *D. bulbifera* (IC_50_ 35.9 ± 2.86 *µ*g/mL) signified similar proteinase inhibitory activity and the plants extract of *N. cadamba* (IC_50_ 60.41 ± 3.88 *µ*g/mL) and *C. arietium* (IC_50_ 60.43 ± 1.71 *µ*g/mL) reported a similar type of antiarthritic activity (Table 5).

## 4. Discussion

The phenolic content of purple-fleshed, orange-fleshed Beauregard, and white-fleshed Bonita sweet potato leaves was estimated by Su et al. 2019 using the Folin–Ciocalteu method. The leaves were extracted using 70% acetone and were diluted to 12.5–200 *μ*g/mL. Also, the result at the highest concentration was found to be in 36.8 ± 4.8 mg GAE/gm in the leaves of purple-fleshed, 41.2 ± 5.0 mg GAE/gm in Beauregard, and 46.7 ± 2.1 mg GAE/gm in Bonita. Comparing the result with the present study of ethanolic leaf extract of *I. batatas* shows less phenolic content i.e. 30.30 ± 0.27 *µ*g GAE/mg than the previous study [43]. Also, Jyothi et al. 2019 estimated that the total phenolic in ethanolic leaves extract of *C. esculenta* was 26.5 mg GAE/g at 1000 *μ*g/mL concentration. The present study result revealed that total phenolic content was highest, i.e., 51.73 ± 0.14 *µ*g GAE/mg compared to previous studies at the same concentration [44].

The total flavonoid content of methanolic extract of *A. viridis* leaves was estimated by Salvamani et al. and Sarker and Oba, which was found to be 152.12 mg rutin equivalent/gm and 182.46 ± 0.26 mg rutin equivalent/gm, respectively. In the present study of ethanolic extract of leaves of *A. viridis*, the total flavonoid content is 23.708 ± 0.81 *µ*g GAE/mg. This shows that the flavonoid content is lower than the previous studies [45, 46].

Ooko Abong et al. researched phytochemical screening in leaves and roots of various sweet potato species. They determined the total tannin content for which 0.15 gm and 0.25 gm of the freeze-dried powdered leaves and roots were taken, and values were represented as mean±standard deviation. Also, the species of sweet potato (K/KA/2004/205) had the highest tannin content of 5.05 gm/100 gm in leaves (40 times) as compared to roots. The tannin content estimated in the present study in the leaf extract of *I. batatas* is 48.50 ± 1.045 *µ*g TAE/mg, which is lesser than the previous study [47]. Also, the presence of tannin content in the tuberous extract of *A. paeoniifolius* with 50% ethanol was determined using the standard protocol described by Majumder et al. 2020. The different concentrations of the samples extracts were prepared as 25–400 *µ*g/mL, and at the highest concentration, 400 *µ*g/mL (0.4 mg/mL) of plant extract was found to be 938.95 ± 90.5 mg TAE/gm. In the present study of ethanolic extract of leaves of *A. paeoniifolius* at a concentration of 400 *μ*g/mL, the tannin content is 54.35 ± 1.217 *µ*g TAE/mg. This clarifies that in the species of *A. paeoniifolius*, the presence of tannin content is more in the tuberous extract than in the leaf extract [48].

Oxidative stress is a condition of imbalanced free radicals in the biological system. It is generated as a by-product of oxygen metabolism. The excessive production of these free radicals plays an essential role in the induction of diseases [49]. In the present study, twelve leaf extracts were obtained from different plants and evaluated for the total phenolic, flavonoid, tannin content, antioxidant activity, antiarthritic, and lipoxygenase inhibition activity.

DPPH is a stable nitrogen-centered free radical and accepts an electron or hydrogen radical to become a stable diamagnetic molecule. When DPPH radicals react with reducing agents, DPPH radicals receive an electron or hydrogen radical from an antioxidant, and corresponding hydrazine is formed. The solution becomes purple to yellow color [50]. The IC_50_ value of 42.48 *μ*g/mL for the DPPH scavenging activity of ethanolic extract of *C. arietinum* by Parithy M et al. 2013 was compared with the present investigation for the same plant extract IC_50_ value of 9.41 ± 0.07 *μ*g/mL, which was found to be higher as compared with previous studies [51]. Similarly, the extract of *C. album* by Debski B. et al., 2018 showed 65 ± 8% significant *p* < 0.05 inhibition, and the present study showed 6.13 ± 1.23% inhibition. This comparison resulted in significantly higher inhibitory activities of present plant extract [52].

At physiological pH, sodium nitroprusside spontaneously generates nitric oxide, which reacts with oxygen to form nitrite ions that may be measured using Griess' reagent. Nitrite ion production is decreased due to competition between nitric oxide scavengers and oxygen [53]. The previous activity by Ghosh et al. on the bulb extract of *Dioscorea bulbifera* showed nitric oxide scavenging activity of 49.85 ± 0.16% at the concentration of 1000 *μ*g/mL and 9.72 ± 0.15% for the recent study on leaf extract of the same plant. Thus, it resulted in higher antioxidant activities of leaves than bulbs of *Dioscorea bulbifera* [54]. The nitric oxide scavenging activity was performed by Griess Illosvoy reaction, and values were represented as mean ± SEM. The result by Alam et al. showed that the nitric oxide scavenging activity at the concentration of 2000 *μ*g/mL of ethanolic extract of leaf of *A. tricolor* was 57.4 ± 2.11, and in the present study, the value of the extract of *A. viridis* at the concentration of 2 mg/mL is 9.51 ± 1.21. This verifies that the species *viridis* is better than *tricolor* [55].

Reactive oxygen species are molecules that generate free oxygen radicals, including hydroxyl and superoxide. These molecules are highly reactive and participate in mitochondrial dysfunctions to prevent the production of ATP and promote cellular disruption [56, 57]. The hydrogen peroxide scavenging activity for the extract of *C. album* was done by Devi et al. and revealed that bay leaf extract showed concentration-dependent inhibition at 1000 *µ*g/mL concentration of 72% and had the highest scavenging activity as compared with BHT. In the present study, the scavenging activity at the same concentration was most effective at 11.73 ± 1.58% but concentration-dependent as compared to the previous study [58].

Potassium ferricyanide (Fe^3+^) combines with substances having reduced potential to generate potassium ferrocyanide (Fe^2+^) at 700 nm. The increased absorbance of the reaction mixture indicates increasing reducing power [59]. Candel et al. evaluated the reducing ability of ethanolic extract of *Anthocephalus cadamba* and found a significant positive correlation between ferric reducing antioxidant power (FRAP) and total flavonoids. It also concluded that extracts enriched with flavonoids and phenolics act like ferric-reducing agents and protect from oxidative stress [60]. For the FRAP activity of methanolic extract of seeds, flowers, stem, and leaves of *Amaranthus caudatus,* FRAP reagent was prepared by Karamac et al. and observed in the *Amaranthus* extracts (1000 *µ*g/mL). It showed the highest absorbance in the following order: leaves ≥ flowers >> stem > seeds. Similarly, the present study of FRAP ethanolic extract of the leaf of *A. viridis* (11.61 ± 2.62 *µ*g/mL) shows increasing activity with increasing absorbance, i.e., concentration-dependent [61].

Hydroxyl (OH^∙^) radical is a robust reactive oxygen species. This moiety directly interacts with polyunsaturated fatty acid found in the cell membrane, damaging all the essential components of the cells and destroying the biological system, which promotes different pathological conditions such as cancer [53]. The earlier study of Ghosh et al. on a bulb of *D. bulbifera* at 1000 *μ*g/mL concentration showed the % inhibition of hydroxyl radical by 64.23 ± 1.25% in the ethanolic extract [62]. But the present study on leaves of *D. bulbifera* revealed the percentage hydroxyl radical scavenging activity of 15.57 ± 0.91% at the same concentration, which was found to be better in the leaf than in the bulb extract. The same scavenging activity was performed by thiobarbituric acid (TBA) method described by Sun et al. 2017. It was carried out in sweet potato leaves, which were extracted using ethanol. At 2000 *μ*g/mL concentration, the % hydroxyl scavenging activity was 65.99 ± 2.07, expressed as mean ± standard deviation. In the present study, the leaf extract of *I. batatas* at the same concentration was 40.9 ± 2.92, which is comparatively higher compared to past studies [63].

Reactive oxygen species are formed from various processes, and the metal oxidation reaction is one of them. The transition of metal ions ferrous (Fe^2+^) possesses the generation of free radicals by gaining or losing the electron. Therefore, different plant extracts were used to determine the inhibition capacity of free radicals via metal chelating activity [64]. The ferrous ion chelating activity was carried out using ferrozine by Vu et al. on the methanolic corn extract of *C. esculenta* and was found to be 61.9% [65]. The present study of ethanolic leaf extract of the same plant is 14.61 ± 0.08%. This shows a higher analysis of leaves than the corn of *C. esculenta*. The previous research carried out by Beevi et al. on the root of *R. sativus* using various solvents such as methanol, water, ethyl acetate, and hexane. It showed 32.15%, 28.54%, 21.98%, and 20.86% of chelating activity, respectively. The present study on leaves of *R. sativus* using ethanolic extract showed 4.76 ± 0.68% inhibition at 1 mg/mL of concentration [66].

The basic principle to assess the total antioxidant capacity through phosphomolybdenum assay includes the reduction of Mo (VI) to Mo (V) by the plant extract possessing antioxidant compounds [67]. Khandayataray et al. and Rajeshwari et al. revealed different activities of leaf extract of *C. album* and *B. nigra* at different concentrations of 100 and 200 *µ*g/m [68, 69]. The *C. album* represented that ethyl acetate, methanol, and aqueous extract had the highest antioxidant capacity of 0.812 ± 0.06, 0.891 ± 0.05, and 0.816 ± 0.11, respectively, compared to the present study at the same concentration of 16.3 ± 0.16 while the *B. nigra* leaves extract from acetone showed the least activity of 95.26 ± 07.85 compared with the present (15.79 ± 0.21) study at the same concentration.

A monomeric protein called lipoxygenase (LOX) is produced when hydroperoxidase is produced by oxidizing polyunsaturated fatty acids such as linoleic, linolenic, and arachidonic acid. LOX is widely distributed in many animals, plants, fungi, and cyanobacteria species. The 5-lipoxygenase is derived from the 5-carbon of arachidonic acid and is commonly found in mammalians. The LOX is expressed in many immune, epithelial, and tumor cells. It is crucial for several physiological conditions, including stroke, neurological illness, skin disorders, tumorigenesis, and cardiovascular complications. Most significantly, it is a precursor to inflammation [37, 38]. Sendangratri et al. carried out lipoxygenase inhibition activity of tuber extract of sweet potatoes of different varieties. The tubers were macerated with 70% ethanol for the activity, and their ability to inhibit soybean lipoxygenase was assessed. Also, the result showed that the IC_50_ values of purple, orange, and white sweet potato tuber extracts were 46.09, 52.12, and 63.69 *μ*g/mL, respectively. However, in a recent study, the IC_50_ value of ethanolic extract of leaves of *I. batatas* is 62.94 ± 3.53 *μ*g/mL at a higher extract concentration, i.e., 2000 *μ*g/mL [70]. Also, the in vitro anti-inflammatory activity, i.e., lipoxygenase inhibition of the same species, was observed and found to be showing the IC_50_ value in the given order *spinosus* > *tricolor* > *dubis* > *viridis*, 58.17 ± 3.49, 66.20 ± 2.67, 72.49 ± 3.44, and 73.93 ± 3.38 *μ*g/mL, respectively. The IC_50_ value of *A. viridis* in the present study is 58.99 ± 3.84 *μ*g/mL, which is comparatively higher than the past studies for the same species [71].

Rheumatoid arthritis is a chronic immunological inflammatory condition. It initially affects the small joints of the hand and legs, which commonly start from the figure joints. The continuous development of this condition damages the joint cartilage, tendon, and ligaments, resulting in intense pain. Different antiarthritic drugs are available, but those drugs only showed symptomatic relief, which is unsuitable for the patient [72]. The primary goal was to look at alternative compounds for managing and preventing the onset of arthritis. Previously, Win KC et al. 2018 performed the protein denaturation and bovine serum albumin procedure to investigate the ethanolic extract of *A. cadamba* for its in vitro antiarthritic capabilities. The percentage of arthritis protection was found to be 45.58% at a concentration of 500 *µ*g/mL. In contrast, the present study shows that the rate of arthritis protection is 60.41 ± 3.88%; it concluded that the *Anthocephalus cadamba* has better antiarthritis activity than the *Neolamarckia cadamba,* and various species showed varying activities.

Arthritic responses have been linked to proteinases. Many serine proteinases are found in the lysosomal granules of neutrophils. Leukocyte proteinases significantly influence the development of tissue damage during inflammatory processes. Recent research has demonstrated that numerous flavonoids contributed substantially to several plants' antioxidant and anti-inflammatory properties. Thus, present research clarifies that bioactivity (flavonoids and many more) may influence anti-inflammatory properties [73]. In previous studies, *Brassica nigra* had the proteinase inhibitory capacity. It showed that the increase in the dose (*µ*g/mL) increased the percentage of proteinase inhibition. At 50, 100, 150, 200, and 250 *µ*g/mL, concentration expressed 15.26, 22.14, 25.36, 32.89, and 42.57 percentage inhibition, respectively. *B. nigra* had the flavonoid content responsible for inhibiting trypsin (regulatory of inflammation) enzyme and thus acted as an anti-inflammatory agent [39]. Prior research by Mahajan et al. on methanolic extract of *D. bulbifera* tubers demonstrated a significant % proteinase inhibition of 13.61% at 0.2 mg/mL concentrations. In contrast, the current study shows an inhibition of 35.9% at the same concentration, which is lesser to the previous study [40].

Anti-inflammatory agents mainly inhibit the cyclooxygenase enzyme responsible for synthesizing inflammatory mediators by converting arachidonic acid to prostaglandins. Prostaglandin G2 (PGG2) is converted into PGH2 together by the enzyme peroxidase, which creates long membrane channels. Due to the release of chemical mediators, membrane channel opening caused the release of arachidonic acid from the membrane and converted to prostaglandin. These enzymes' extracellular activity is considered to be related to both acute and chronic inflammation. Sen et al. conducted in vitro anti-inflammatory research in *A. caudatus L* by observing the inhibition of protein denaturation and HRBC membrane stabilizing assay. At a 500 *μ*g/mL concentration, the methanolic extract of leaves from *A. caudatus L* impacted membrane stability and protein denaturation. But at an 800 *μ*g/mL concentration, the ethanolic extract of *A. viridis* exhibits the same activity of membrane stabilization and protein denaturation. Hence, the % inhibition in the present study is comparatively higher than in the past studies [74].

### 4.1. Limitation of the Study

In the present study, we only carried out the in- vitro study of selected plant extracts. Due to limited resources, we were unable to do in- vivo assay, molecular pathway analysis, and quantitative structure-activity relationship (QSAR) modeling of identified compounds through mass spectroscopy. In future, computation and molecular analysis should be carried out for validation of our study.

## 5. Conclusion

*C. arietinum* expressed highest moisture content, and *A. viridis* had highest chlorophyll content. All plant extracts showed good phenolic, flavonoid, and tannin content. While *N. cadamba* and *D. bulbifera* have highest total phenolic content, *I. batatas* and *C. album* have flavonoid highest value. Similarly, *R. sativus* and *N. cadamba* reported highest total tannin content. Also, different phytoconstituents were identified from several plant extracts through gas chromatography and mass spectroscopy (GC-MS) methods. The present study used twelve leaves extracts for the antioxidant and anti-inflammatory activities. This study concluded that all the selected medicinal plants contain a good amount of phytoconstituents and show significant antioxidant and anti-inflammatory activities.

## Figures and Tables

**Table 1:** List of plant names, specimen numbers, percentage yield, moisture, and chlorophyll content of selected plants.

S. no.	Plants extract	Specimen number	Yield (%)	Moisture content (%)	Chlorophyll content (mg/g of sample)
1	*Cicer arietinum*	113/2078/2079	12	84.12 ± 0.011	0.109 ± 0.035
2	*Murraya koenigii*	113/2078/2079	9	68.92 ± 0.033	0.863 ± 0.009
3	*Colocasia esculenta*	113/2078/2079	7	85.69 ± 0.124	2.257 ± 0.001
4	*Amaranthus viridis*	118/2078/2079	7.8	95 ± 0.026	2.8254 ± 0.028
5	*Ipomoea batatas*	118/2078/2079	13.2	85 ± 0.022	1.5518 ± 0.004
6	*Amorphophallus paeoniifolius*	118/2078/2079	8.2	77.41 ± 0.070	13.5947 ± 0.003
7	*Raphanus sativus*	120/2078/2079	15.05	91.003 ± 0.017	1.669 ± 0.019
8	*Neolamarckia cadamba*	120/2078/2079	17.62	61.242 ± 0.041	5.472 ± 0.005
9	*Dioscorea bulbifera*	120/2078/2079	13.83	75.587 ± 0.079	2.173 ± 0.011
10	*Chenopodium album*	117/2078/2079	9	0.5 ± 0.005	0.245 ± 0.001
11	*Cinnamomum tamala*	117/2078/2079	14	49.9 ± 0.015	0.364 ± 0.001
12	*Brassica nigra*	117/2078/2079	12	27.8 ± 0.029	0.216 ± 0.035

*Note.* Each value in above table is represented as mean ± SEM (*n* = 3).

**Table 2:** Preliminary phytochemical screening of twelve sessional medicinal plants.

S. no.	Plants name	Alkaloids				Saponin	Tannin	Flavonoid	Vit. C	Oxalate	Phenolic	Glycoside	Phytate
		Mayer's test	Dandruff's test	Hager's test	Wagner's test	Froth test					Lead acetate test	Liebermann–Burchard test	
1	*C. arietinum*	+	+	−	+	−	+	+	^*∗*^	^*∗*^	+	−	^*∗*^
2	*M. koenigii*	+	+	+	−	−	+	+	^*∗*^	^*∗*^	+	−	^*∗*^
3	*C. esculenta*	+	+	+	−	+	+	+	^*∗*^	^*∗*^	+	+	^*∗*^
4	*A. viridis*	+	+	+	+	−	^*∗*^	+	+	−	+	−	−
5	*I. batatas*	+	+	+	+	+	^*∗*^	+	+	−	+	−	−
6	*A. paeoniifolius*	+	+	+	+	−	^*∗*^	+	+	+	+	+	+
7	*R. sativus*	+	+	−	−	−	+	−	+	^*∗*^	+	−	^*∗*^
8	*N. cadamba*	+	−	+	+	+	+	+	+	^*∗*^	+	−	^*∗*^
9	*D. bulbifera*	+	+	+	−	+	+	+	+	^*∗*^	+	+	^*∗*^
10	*C. album*	+	+	−	−	−	+	+	−	−	+	−	−
11	*C. tamala*	+	+	+	−	−	+	+	+	^*∗*^	+	−	^*∗*^
12	*B. nigra*	+	+	+	−	−	+	+	−	−	+	+	−

− indicates absence of phytoconstituents, + indicates presence of phytoconstituents, and ^*∗*^indicates no determination of phytoconstituents.

**Table 3:** Bioactive compounds present in different leaves plant extracts detected through GC-MS analysis.

S. no.	RT (min)	Peak width 50% (min)	Compound name
*Cicer arientinium*			
1	37.970	6.92	5,10-Octacosanediol
2	38.607	7.16	Globulol
3	41.261	8.55	7,10-Octadecadienoic acid, methyl ester
4	41.660	9.05	Reticuline, 6′-methyl
5	41.778	51.97	9,12,15-Octagecatrienoic acid ethyl ester
6	52.945	5.36	2-p-Nitrophenyl-oxadiazol-1,3,4-one-5
7	54.908	5.36	1,4-Phthalazinedione, 2,3-dihydro-6-nitro-methyltris (trimethylsiloxy) silane
8	58.123	5.31	1,4-Phthalazinedione, 2,3-dihydro-6-nitro cyclotrisiloxane

*Colcasia esculenta*			
1	29.663	7.98	Ethyl decanoate
2	37.981	16.68	(+)-Valencene
3	41.250	14.68	cis-Vaccenic acid
4	41.660	9.53	Reticuline, 6′-methyl
5	53.020	7.85	Cyclotrisiloxane, hexamethyl-4-methyl-2-trimethylsilyloxy-acetophenone
6	53.721	10.42	Cyclotrisiloxane, hexamethyl-cyclotrisiloxane, hexamethyl-2-(acetomethyl)-3-(methoxycarbonyl) biphenylene
7	53.916	8.12	Cyclotrisiloxane, hexamethyl-benzo (h) quinoline, 2,4-dimethyl-1,2-benzenediol, 3, 5-bis (1,1-dimethylethyl)-
8	54.207	10.50	Cyclotrisiloxane, hexamethyl-silicic acid, dimethyl bis (trimethyl lsilyl) ester

*Amaranthus viridis*			
1	2.151	55.10	(2E,4Z)-Hexadiene
2	28.389	24.93	Phenol, 2,4-bis (1,1-dimethylethyl)
3	37.981	19.96	(+)-Valencene

*Ipomoea batatas*			
1	37.981	4.36	Stigmasterol
2	51.035	3.16	Tetrasiloxane, decamethyl-
3	53.603	3.34	Cyclotrisiloxane, hexamethyl-
4	53.961	3.86	Cyclotrisiloxane, hexamethyl-methanol, [4-(1,1-dimethylethyl) phenoxy]-, acetate
5	54.110	3.46	Trimethyl [4-(1,1,3,3,-tetramethyl beutyl) phenoxy] silane
6	54.412	3.10	Silane, 1,4-phenylenebis [trimethyl cyclotrisiloxane, hexamethyl-1H-indole, 1-,ethyl-2-phenyl
7	54.67	4.65	Cyclotrisiloxane, hexamethyl-benzo [h] quinoline, 2,4-dimethyl-trimethyl [4-(2-methyl-4-oxo-2-pentyl) phenoxy] silane
8	56.386	3.48	Cyclotrisiloxane, hexamethyl-
9	56.742	3.50	Cyclotrisiloxane, hexamethyl-4-methyl-2-trimethylsiloxy-aceto phenone
10	57.853	3.26	1,4-Phenylenebis [trimethyl]
11	58.544	2.90	1,2-Benzendiol, 3,5-bis (1,1-dimethylethyl)
12	59.126	2.84	Tetrasiloxane, decamethyl
13	59.267	3.92	Tetrasiloxane, hexamethyl-methanol
14	59.375	6.24	Cyclotrisiloxane, hexamethyl-silane

*Amorphophallus paeoniifolius*			
1	37.301	14.36	Tridecanoic acid
2	41.045	19.64	Octadecanoic acid
3	53.937	4.49	1,4-Phthalazinedione, 2,3-dihydro-6-nitro-cyclotrisiloxane
4	54.466	4.55	cis-4-Ethoxy-b-methyl-b-nitrostyrene
6	54.692	6.08	Tetrasiloxane, decamethyl
7	54.919	5.61	4,6-Di-tert-butylresorcinol

*Raphanus sativus*			
1	28.390	45.74	Phenol. 2.4-bis (1,1-dimethylethyl)
2	54.131	28.67	Cyclotrisiloxane, hexamethyl-

*Neolamarckia cadamba*			
1	38.606	17.75	Hexadecenoic acid, ethyl ester
2	52.006	8.91	2,4,6-Cycloheptatrien-1-one, 3,5-bis-trimethylsilyl
3	53.117	3.77	Cyclotrisiloxane, hexamethyl
4	53.905	4.79	Tetrasiloxane, decamethyl
5	54.649	6.46	4-Methyl-2-trimethylsilyoxy-acetophenone
6	54.930	4.50	2,4,6-Cycloheptatrien-1-one, 3,5,-bis-trimethylsilyl
7	55.588	3.47	1,2-Benzenediol, 3,5-bis (1,1-dimethyethyl)-
8	57.691	3.36	Benzo [h] quinoline, 2,4-dimethyl
9	59.385	6.86	1H-Indole, 1-methyl-2-phenyl

*Dioscorea bulbifera*			
1	2.152	14.29	2-Pentanone, 4-hydroxy-4-meth
2	40.797	9.62	3,5‐Dimethyl‐1‐ hexylpyrazole
3	41.778	28.16	Curcuphenol
4	54.077	13.66	Cyclotrisiloxane, hexamethyl
5	55.059	14.57	1,4-Phthalazinedione, 2,3-dihydro-6-nitro

*Chenopodium album*			
1	2.141	15.20	Unknown
2	2.443	4.75	N-Demethylpromethazine
3	37.301	5.87	Tetratriacontane
4	38.002	6.72	9-Octadecenoic acid (Z) methyl ester
5	40.559	5.69	*α*-Linolenic acid
6	41.660	8.88	Reticuline, 6′-methyl
7	41.778	24.66	9,12,15-Octadecatrienoic acid, ethyl ester
8	52.265	6.24	1-Methyl-3-phenylindole
9	56.354	5.41	Cyclotrisiloxane, hexamethyl
10	57.120	5.66	Tetrasiloxane, decamethyl
11	57.843	6.11	1H-Indole, 1-methyl-2-phenyl
12	58.188	4.82	1,4-Phenylenebis, trimethyl

*Cinnamomum tamala*			
1	2.141	13.81	Octopamine (p-hydroxyphenylethanolamine)
2	38.606	7.93	Unknown
3	40.786	7.98	Unknown
4	41.778	22.04	Unknown
5	52.977	3.90	1,4-Phthalazinedione, 2,3-dihydro-6-nitro silicic acid
6	53.193	5.67	Tetrasiloxane, decamethyl
7	53.441	3.90	1,2-Benzisothiazol-3-amine
8	54.077	7.13	2,4,6-Cycloheptatrien-1-one, 3,5-bis-trimethylsilyl
9	54.886	4.15	1,2-Benzisothiazol-3-amine
10	56.181	6.05	1-Methyl-3-phenylindole
11	56.742	3.82	2-(Acetoxymethyl)-3-(methoxycarbonyl) biphenylene
12	58.296	4.23	Cyclotrisiloxane, hexamethyl
13	59.126	4.10	Benzo [h] quinoline, 2,4-dimethyl
14	59.288	3.93	Cyclotrisiloxane, hexamethyl

**Table 4:** Antioxidant activity of twelve sessional medicinal plants.

Plant names	Nitric oxide	H_2_O_2_	DPPH	Ferrous ion	Hydroxyl radical	Ferric reducing	Total antioxidant
Ascorbic acid	3.91 ± 0.80	3.31 ± 0.58	4.54 ± 0.98	3.68 ± 0.59	4.75 ± 0.43	2.47 ± 0.40	2.87 ± 0.10
BHT	—	2.89 ± 0.32	2.90 ± 0.51	2.81 ± 0.82	2.90 ± 0.98	—	—
BHA	5.58 ± 0.68	—	—	—	—	3.02 ± 0.30	—
*N. cadamba*	15.11 ± 1.29	7.01 ± 0.14	7.91 ± 1.13	29.441 ± 0.22	17.52 ± 0.83	11.49 ± 0.98	11.76 ± 0.35
*D. bulbifera*	9.72 ± 0.15	13.95 ± 0.17	16.65 ± 0.24	15.63 ± 0.96	15.57 ± 0.91	17.38 ± 0.71	28.28 ± 0.13
*R. sativus*	13.39 ± 0.11	6.89 ± 0.16	5.84 ± 0.14	4.76 ± 0.68	16.13 ± 0.54	14.97 ± 2.06	8.99 ± 0.20
*C. arietinum*	9.06 ± 0.10	12.66 ± 0.03	9.41 ± 0.07	16.08 ± 0.21	27.82 ± 1.88	31.31 ± 1.72	8.38 ± 0.09
*C. esculenta*	13.29 ± 0.03	7.56 ± 0.52	8.91 ± 0.12	14.61 ± 0.08	7.22 ± 0.56	33.84 ± 1.90	14.46 ± 0.11
*M. koenigii*	16.79 ± 0.21	17.14 ± 0.61	16.68 ± 0.04	18.62 ± 0.66	7.12 ± 0.66	45.41 ± 1.65	16.24 ± 1.34
*B. nigra*	16.21 ± 1.61	8.92 ± 1.36	8.44 ± 0.97	15.23 ± 1.95	15.27 ± 1.86	15.98 ± 0.97	15.79 ± 0.21
*C. tamala*	35.06 ± 0.99	7.81 ± 1.8	25.02 ± 1.14	14.89 ± 1.25	32.59 ± 1.47	12.39 ± 2.62	31.37 ± 1.44
*C. album*	29.8 ± 0.17	11.73 ± 1.58	6.13 ± 1.23	9.62 ± 0.30	43.54 ± 0.19	15.61 ± 0.97	16.3 ± 0.16
*A. viridis*	9.51 ± 1.21	31.07 ± 2.78	8.70 ± 0.39	11.28 ± 2.61	39.56 ± 0.25	11.61 ± 2.62	12.39 ± 1.97
*I. batatas*	18.56 ± 1.82	6.63 ± 0.46	11.65 ± 2.76	24.30 ± 3.03	40.95 ± 2.92	10.07 ± 1.48	10.6 ± 0.18
*A. paeoniifolius*	12.06 ± 0.60	29.11 ± 2.69	9.56 ± 1.54	54.44 ± 3.68	44.68 ± 1.15	11.12 ± 1.52	13.43 ± 2.45

**Table 5:** Anti-inflammatory and antiarthritic activities of twelve sessional medicinal plants.

Plant names	Proteinase inhibition	Protein denaturation	Antiarthritic	5-LOX	HRBC
Indomethacin	8.63 ± 1.97	6.28 ± 0.65	11.25 ± 2.84	7.24 ± 0.23	7.14 ± 0.31
Aceclofenac	18.13 ± 1.25	7.83 ± 0.93	13.28 ± 0.23	11.85 ± 0.67	21.90 ± 2.34
Etoricoxib	17.26 ± 1.82	8.20 ± 0.35	26.78 ± 2.63	15.32 ± 1.75	10.97 ± 1.65
Aspirin	15.28 ± 1.92	11.95 ± 1.40	14.90 ± 0.12	15.31 ± 1.95	21.28 ± 0.43
*N. cadamba*	35.98 ± 2.99	62.11 ± 2.50	60.41 ± 3.88	46.21 ± 3.39	16.05 ± 2.67
*D. bulbifera*	35.9 ± 2.86	36.83 ± 2.92	66.90 ± 4.03	15.01 ± 0.23	15.69 ± 2.63
*R. sativus*	65.52 ± 2.53	7.48 ± 0.48	21.03 ± 2.73	49.64 ± 3.52	9.72 ± 1.12
*C. arietinum*	29.45 ± 1.46	61.65 ± 2.67	60.43 ± 1.71	14.67 ± 1.94	19.36 ± 2.55
*C. esculenta*	13.50 ± 2.94	44.72 ± 3.34	56.2 ± 3.74	48.21 ± 3.32	49.23 ± 3.07
*M. koenigii*	28.57 ± 2.39	57.29 ± 4.27	47.93 ± 2.16	42.37 ± 2.35	59.14 ± 2.85
*B. nigra*	13.1 ± 1.81	40.79 ± 3.19	21.87 ± 2.13	17.5 ± 2.09	14.85 ± 1.92
*C. tamala*	46.57 ± 3.41	52.78 ± 6.36	11.81 ± 2.51	68.16 ± 4.34	30.60 ± 2.76
*C. album*	23.47 ± 2.42	20.51 ± 2.26	9.44 ± 1.62	68.89 ± 2.35	70.72 ± 4.20
*A. viridis*	34.85 ± 3.15	15.09 ± 1.94	55.57 ± 3.72	58.99 ± 3.84	20.65 ± 0.91
*I. batatas*	17.22 ± 0.50	22.86 ± 2.39	61.27 ± 3.91	62.94 ± 3.53	10.26 ± 0.65
*A. paeoniifolius*	23.18 ± 1.48	28.51 ± 2.67	62.36 ± 3.94	63.96 ± 3.99	6.22 ± 0.78

## Data Availability

The data and material that were used to support the present study are included in this manuscript.

## Supplementary Materials

Supplementary Materials Total phenolic content, total flavonoid content, total tannin content, and GC-MS spectra are available in a supplementary file.
