# Supplementary material for: Evaluation of Antioxidant and Anti-Inflammatory Activities, and Metabolite Profiling of Selected Medicinal Plants of Nepal
Source: J Trop Med. 2023 Nov 3;2023:6641018. doi: 10.1155/2023/6641018 (PMC10637841; doi:10.1155/2023/6641018)
Supplement: Supplementary Materials — Total phenolic content, total flavonoid content, total tannin content, and GC-MS spectra are available in a supplementary file. [file 6641018.f1.docx]

**Evaluation of Antioxidant and Anti-Inflammatory Activities, and Metabolite Profiling of Selected Medicinal Plants of Nepal**

Amit Kumar Shrivastava*^1^, Muskan Keshari^2^, Manisha Neupane^2^, Shesbhan Chaudhary^2^, Purna Kala Dhakal^2^, Laxmi Shrestha^1^, Anjan Palikhey^1^, Chandrajeet Kumar Yadav^1^, Gopal Lamichhane^3^, Mohammad Ujair Shekh^4^, Rakesh Kumar Yadav^5^,

^1^Department of Pharmacology, Universal College of Medical Sciences, Bhairahawa, Rupandehi, Nepal, 32900

^2^Department of Pharmacy, Universal College of Medical Sciences, Bhairahawa, Rupandehi, Nepal, 32900

^3^Department of Neutritional Sciences, Oklahoma State University, Stillwater, OK, 74078, USA.

^4^School of Health and Allied sciences, Pokhara University, Pokhara-30, Kaski, Nepal

^5^Institute of Agriculture and Animal Science, Tribhuvan University, Paklihawa, Rupandehi, Nepal

Corresponding author

Amit Kumar Shrivastava

Universal College of Medical Sciences, Bhairahwa, Rupandehi, Nepal

Email: sr.akshri.ucms.np@gmail.com

**Abbreviations**

DPPH 2,2-Diphenyl-1-picrylhydrazyl

IC_50_ Inhibitory concentration (50%)

HRBC Human Read Blood Cell
GC-MS Gas Chromatography and Mass Spectroscopy

(IL)-1*β* Interleukin-1*β*

IL Interleukin

(TNF)-α Tumor Necrosis Factor-α

IL-1Rα Interleukin-1 Receptor- α

DNA Deoxyribose Neucleic Acid

RNA Ribose Neucleic Acid

ROS Reactive Oxygen Species

MAPK mitogen-activated protein kinases

CXC Chemokine Receptor

℃ Degree Celsius

gm Gram

mg Milligram

% Percentage

MC Moisture content

PVP Polyvinylpolypyrrolidone

rpm Revolution Per Minute

TPC Total Phenolic Content

TFC Total Flavonoid Content

TAE Tannin Content Equivalent

GAE Gallic Acid Equivalent

UV spectrophotometer Ultra Violet Spectrophotometer

*µ*L Microliter

Fe^2+^ Ferrous

Fe^3+^ Ferric

NO Nitric Oxide

OH Hydroxyl

H_2_O_2_ Hydrogen Peroxide

eV Electric Volt

DMSO Dimethyl Sulfoxide

BSA Bovine Serum Albumin

PBS Phosphate Buffer Saline

RBC Red Blood Cell

Tris-HCl Tris-Hydrochloride

M Mole

U/mL Unit/milliliter

Mmol Millimol

SEM Standard Error Mean

mg/mL Milligram/Milliliter

QAE Quercetin Equivalent

BHT Butylated Hydroxyl Toulene

BHA Butylated Hydroxyl Annisole

LOX Lipoxygenase

FRAP Ferric Reducing Anti-oxidant Power

TBA Thiobarbituric Acid

*C. arietinum Cicer arietinum*

*M. koiengii Murraya koiengii*

*C. esculenta Colocasia esculenta*

*A. viridis Amaranthus viridis*

*I. batatus Ipomoea batatus*

*A. paeoniifolius Amorphophallus paeoniifolius*

*R. sativus Raphanus sativus*

*N. cadamba Neolamarckia cadamba*

*D. bulbifera Dioscorea bulbifera*

*C. album Chenopodium album*

*C. tamala Cinnamomum tamala*

*B. nigra Brassica nigra*

| **Supplementary table 1: Total phenolic content of twelve sessional medicinal plants.** | | | | | | | | | | |
| --- | --- | --- | --- | --- | --- | --- | --- | --- | --- | --- |
| **Plant extract** | **Mean Absorbance 765nm** | | | **Concentration equivalent to Gallic acid (*µ*g/mL)** | | | **Total phenolic content (*µ*gGAE/mg)** | | | **Total (*µ*gGAE/mg)** |
| *C. arietinum* | 0.134±0.03 | 0.173±0.01 | 0.173±0.01 | 13.13±0.24 | 17.01±0.04 | 17.01±0.17 | 131.32±0.04 | 170.10±0.03 | 170.10±0.17 | 157.17±0.70 |
| *M. koiengii* | 0.038±0.01 | 0.041±0.03 | 0.037±0.03 | 3.23±0.24 | 3.59±0.02 | 3.13±0.26 | 32.34±0.36 | 35.91±0.16 | 31.32±0.12 | 33.19±2.41 |
| *C. esculenta* | 0.056±0.03 | 0.052±0.01 | 0.057±0.01 | 5.07±0.34 | 4.71±0.04 | 5.17±0.67 | 50.71±0.23 | 47.14±0.10 | 51.73±0.14 | 49.86±2.41 |
| *A. viridis* | 0.163±0.01 | 0.169±0.05 | 0.164±0.03 | 15.99±0.23 | 16.65±0.17 | 16.14±0.14 | 159.89±0.02 | 166.53±0.27 | 161.42±0.31 | 162.61±3.48 |
| *I. batatus* | 0.031±0.00 | 0.036±0.01 | 0.034±0.01 | 2.57±0.13 | 3.03±0.59 | 2.87±0.31 | 25.71±0.49 | 30.30±0.27 | 28.77±0.24 | 28.26±2.34 |
| *A. paeoniifolius* | 0.104±0.02 | 0.104±0.01 | 0.106±0.01 | 9.96±0.10 | 10.02±0.34 | 10.22±0.17 | 99.69±0.56 | 100.20±0.16 | 102.24±0.33 | 100.71±1.35 |
| *R. sativus* | 0.155±0.01 | 0.156±0.00 | 0.154±0.01 | 15.22±0.18 | 15.27±0.38 | 15.07±0.26 | 152.24±0.16 | 152.75±0.15 | 150.71±0.11 | 151.9±1.06 |
| *N. cadamba* | 0.235±0.05 | 0.243±0.00 | 0.226±0.00 | 23.38±0.15 | 24.15±0.45 | 24.15±0.35 | 233.87±0.24 | 241.53±0.20 | 224.18±0.06 | 233.19±8.69 |
| *D. bulbifera* | 0.198±0.01 | 0.207±0.03 | 0.205±0.01 | 19.56±0.18 | 20.48±0.14 | 20.32±0.15 | 195.61±0.09 | 204.79±0.19 | 203.26±0.03 | 201.22±4.92 |
| *C. album* | 0.064±0.01 | 0.066±0.00 | 0.064±0.00 | 5.93±0.08 | 6.09±0.50 | 5.93±0.21 | 59.38±0.31 | 60.91±0.30 | 59.38±0.27 | 59.89±0.88 |
| *C. tamala* | 0.165±0.00 | 0.171±0.01 | 0.165±0.00 | 16.19±0.03 | 16.85±0.33 | 16.19±0.11 | 161.93±0.17 | 168.57±0.32 | 161.93±0.20 | 164.14±3.83 |
| *B. nigra* | 0.165±0.00 | 0.167±0.00 | 0.167±0.00 | 16.24±0.04 | 16.39±0.17 | 16.44±0.02 | 162.44±0.14 | 163.98±0.13 | 164.49±0.12 | 163.63±1.07 |
| **Note:** Each value in above table is represented as Mean ± SD, dilution factor: 10 | | | | | | | | | | |

| **Supplementary table 2: Total flavonoid content of twelve sessional medicinal plants.** | | | | | | | | | | |
| --- | --- | --- | --- | --- | --- | --- | --- | --- | --- | --- |
| **Plant extract** | **Mean Absorbance 765nm** | | | **Concentration equivalent to quercetin (*µ*g/mL)** | | | **Total flavonoid content (*µ*gQAE/mg)** | | | **Total (*µ*gQAE/mg)** |
| *C. arietinum* | 0.128±0.00 | 0.127±0.01 | 0.127±0.00 | 2.58±0.12 | 2.56±0.18 | 2.54±0.11 | 25.82±1.56 | 25.61±0.76 | 25.40±1.24 | 25.61±0.21 |
| *M. koiengii* | 0.125±0.02 | 0.128±0.00 | 0.126±0.00 | 2.50±0.14 | 2.57±0.20 | 2.55±0.17 | 25.08±1.48 | 25.72±1.25 | 25.50±1.33 | 25.44±0.32 |
| *C. esculenta* | 0.189±0.01 | 0.178±0.03 | 0.198±0.03 | 3.86±0.23 | 3.64±0.26 | 4.66±0.13 | 38.64±0.97 | 36.41±1.38 | 46.69±0.92 | 40.58±5.40 |
| *A. viridis* | 0.118±0.00 | 0.114±0.01 | 0.116±0.00 | 2.37±0.69 | 2.28±0.23 | 2.29±0.36 | 23.70±0.81 | 22.86±1.62 | 22.96±0.73 | 23.18±0.46 |
| *I. batatus* | 0.257±0.00 | 0.253±0.02 | 0.251±0.01 | 5.30±0.11 | 5.22±0.43 | 5.01±0.23 | 53.05±1.11 | 52.20±1.24 | 50.19±0.47 | 51.82±1.47 |
| *A. paeoniifolius* | 0.175±0.03 | 0.176±0.00 | 0.176±0.00 | 3.57±0.14 | 3.60±0.31 | 3.61±0.47 | 35.78±1.37 | 35.99±1.88 | 36.10±1.31 | 35.96±0.16 |
| *R. sativus* | 0.145±0.01 | 0.142±0.00 | 0.144±0.00 | 2.93±0.22 | 2.87±0.35 | 2.93±0.24 | 29.32±0.53 | 28.79±1.23 | 29.32±1.69 | 29.15±0.31 |
| *N. cadamba* | 0.166±0.00 | 0.165±0.00 | 0.165±0.00 | 3.38±0.17 | 3.36±0.61 | 3.36±0.13 | 33.87±1.82 | 33.66±1.43 | 33.66±1.24 | 33.74±0.12 |
| *D. bulbifera* | 0.162±0.01 | 0.170±0.00 | 0.169±0.01 | 3.30±0.47 | 3.60±0.49 | 3.46±0.78 | 33.03±1.71 | 35.99±1.40 | 34.61±0.87 | 34.55±1.48 |
| *C. album* | 0.216±0.05 | 0.216±0.01 | 0.271±0.00 | 4.44±0.30 | 4.48±0.38 | 4.44±0.54 | 44.47±0.67 | 44.89±1.18 | 44.47±0.91 | 44.61±0.24 |
| *C. tamala* | 0.114±0.00 | 0.115±0.00 | 0.116±0.00 | 2.27±0.18 | 2.41±0.22 | 2.30±0.27 | 22.75±1.38 | 24.13±1.14 | 23.07±1.36 | 23.32±0.72 |
| *B. nigra* | 0.069±0.00 | 0.062±0.00 | 0.060±0.01 | 1.33±0.56 | 0.89±0.44 | 1.17±0.46 | 13.32±1.50 | 8.98±1.38 | 11.73±1.30 | 11.35±2.20 |
| **Note:** Each value in above table is represented as Mean ± SD, dilution factor: 10 | | | | | | | | | | |

| **Supplementary table 3: total tannin content** **of twelve sessional medicinal plants.** | | | | | | | | | | |
| --- | --- | --- | --- | --- | --- | --- | --- | --- | --- | --- |
| **Plant extract** | **Mean Absorbance (700 nm)** | | | **Concentration equivalent to tannic acid (*µ*g/mL)** | | | **Total tannin content (*µ*g/TAE/mg)** | | | **Total (*µ*g/TAE/mg)** |
| *C. arietinum* | 0.082±0.005 | 0.088±0.002 | 0.086±0.001 | 5.14±0.142 | 5.5±0.091 | 5.40±0.032 | 51.42±0.940 | 55±0.409 | 54.02±1.846 | 53.48±1.85 |
| *M. koiengii* | 0.055 ±0.007 | 0.058±0.002 | 0.055±0.001 | 3.38±0.271 | 3.55±0.038 | 3.38±0.026 | 33.89±1.620 | 35.51±1.168 | 33.89±0.937 | 34.43±0.94 |
| *C. esculenta* | 0.055±0.003 | 0.052±0.002 | 0.053±0.002 | 3.38±0.102 | 3.16±0.017 | 3.22±0.047 | 33.89±0.316 | 31.62±1.117 | 32.27±1.170 | 32.59±1.17 |
| *A. viridis* | 0.087±0.003 | 0.083±0.001 | 0.082±0.00 | 5.46±0.038 | 5.20±0.021 | 5.11±0.059 | 54.67±1.136 | 52.07±1.105 | 51.10±1.846 | 52.61±1.85 |
| *I. batatus* | 0.073±0.004 | 0.078±0.002 | 0.073±0.004 | 4.52±0.115 | 4.85±0.011 | 4.52±0.012 | 45.25±1.521 | 48.50±1.045 | 45.25±1.874 | 46.33±1.88 |
| *A. paeoniifolius* | 0.087±0.004 | 0.081±0.002 | 0.084±0.00 | 5.43±0.204 | 5.04±0.235 | 5.24±0.182 | 54.35±1.217 | 50.45±0.921 | 52.40±1.948 | 52.40±1.95 |
| *R. sativus* | 0.108±0.002 | 0.106±0.004 | 0.101±0.001 | 6.79±0.145 | 6.66±0.087 | 6.37±0.076 | 67.98±1.014 | 66.68±1.461 | 63.76±2.161 | 66.14±2.16 |
| *N. cadamba* | 0.113±0.007 | 0.118±0.002 | 0.114±0.006 | 7.15±0.012 | 7.44±0.016 | 7.18±0.095 | 71.55±2.109 | 74.48±1.081 | 71.88±1.601 | 72.64±1.60 |
| *D. bulbifera* | 0.112±0.001 | 0.114±0.001 | 0.115±0.002 | 7.09±0.156 | 7.22±0.125 | 7.25±0.110 | 70.90±1.098 | 72.20±1.116 | 72.53±0.859 | 71.88±0.86 |
| *C. album* | 0.093±0.003 | 0.100±0.005 | 0.093±0.003 | 5.85±0.031 | 6.311±0.121 | 5.85±0.082 | 58.57±1.102 | 63.11±1.501 | 58.57±2.624 | 60.08±2.62 |
| *C. tamala* | 0.092±0.003 | 0.097±0.009 | 0.102±0.003 | 5.79±0.026 | 6.11±0.044 | 6.44±0.077 | 57.92±1.055 | 61.16±1.135 | 64.41±3.246 | 61.16±3.25 |
| *B. nigra* | 0.104±0.006 | 0.103±0.016 | 0.109±0.003 | 6.53±0.132 | 6.47±0.251 | 6.89±0.056 | 65.38±1.148 | 64.74±1.106 | 68.96±2.277 | 66.36±2.27 |
| **Note:** Each value in above table is represented as Mean ± SD, dilution factor: 10 | | | | | | | | | | |

**Description of the GC-MS Spectra**

The medicinal plants were used in this study were available at surroundings. Also, few of the plants are grown in winter season. From the selected plants are used as vegetables. Objective of the present study was to determine the phytoconstituent of different ethanolic leaves extract. In supplementary figure 1 (S1): expressed GC-MS spectra of the different leaves extracts. These spectra help to determine and compare the presence of phytoconstituents present in different plants. This list of phytoconstituents of different GC-MS spectra was listed in the main manuscript.


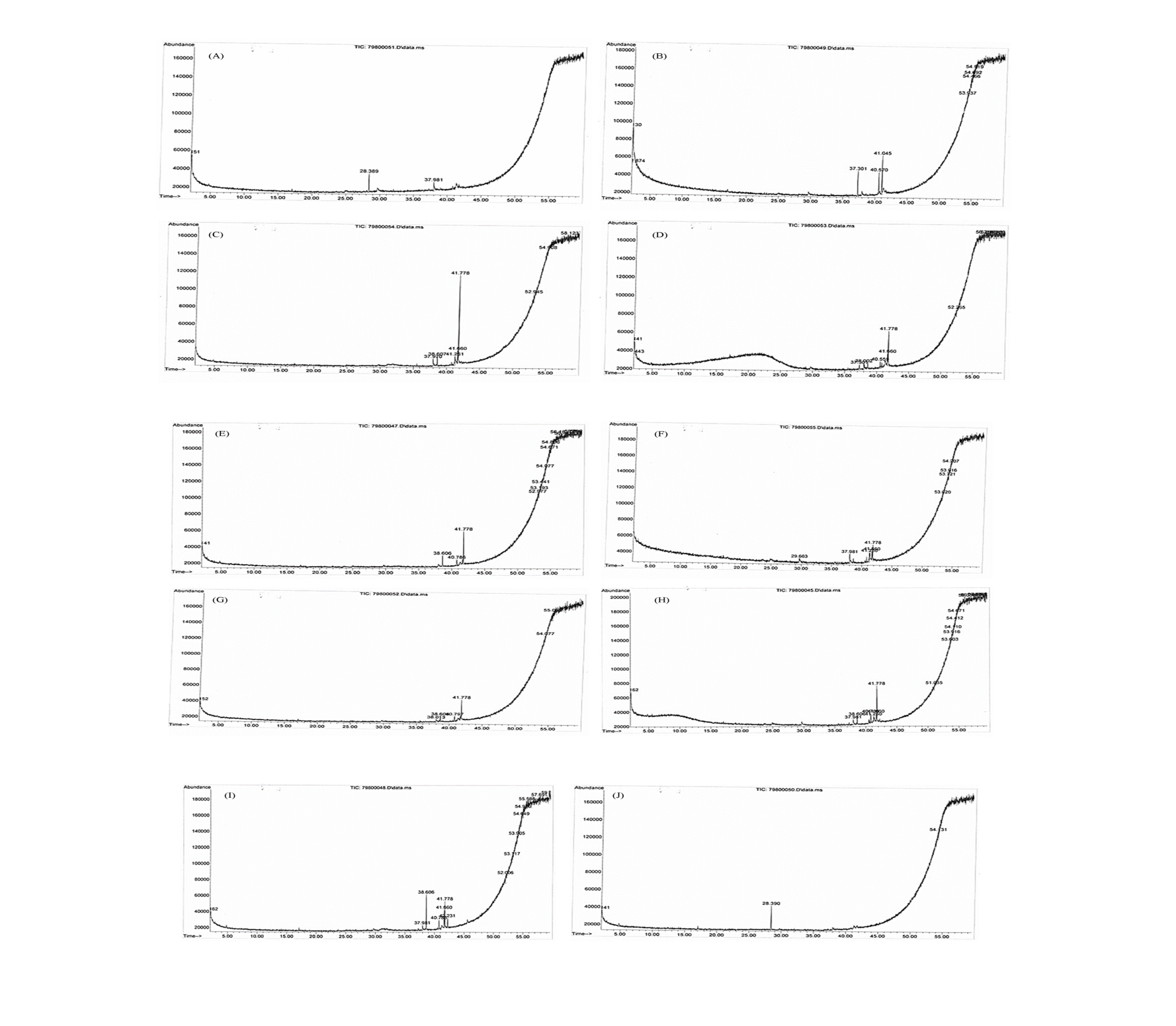
**Supplementary figure 1:** GC-MS spectrum of selected plant leaves extracts (A) *Amaranthus viridis* (B) *Amorphophallus paeoniifolius* (C) *Cicer arietinum* (D) *Chenopodium album* (E) *Cinnamomum tamala* (F) *Colocasia esculenta* (G) *Dioscorea bulbifera* (H) *Ipomoea batatus* (I) *Neolamarckia cadamba* (J) *Raphanus sativus.*
